# Butyrate Attenuates Lung Inflammation by Negatively Modulating Th9 Cells

**DOI:** 10.3389/fimmu.2019.00067

**Published:** 2019-01-29

**Authors:** Raquel de Souza Vieira, Angela Castoldi, Paulo José Basso, Meire Ioshie Hiyane, Niels Olsen Saraiva Câmara, Rafael Ribeiro Almeida

**Affiliations:** ^1^Laboratory of Transplantation Immunobiology, Department of Immunology, Institute of Biomedical Sciences, University of São Paulo, São Paulo, Brazil; ^2^Nephrology Division, Department of Medicine, Federal University of São Paulo, São Paulo, Brazil; ^3^Renal Pathophysiology Laboratory, Department of Clinical Medicine, University of São Paulo, São Paulo, Brazil; ^4^Laboratory of Immunology, Heart Institute (InCor) School of Medicine, University of São Paulo, São Paulo, Brazil

**Keywords:** butyrate, Th9 cells, tregs, eosinophils, lung inflammation

## Abstract

Th9 cells orchestrate allergic lung inflammation by promoting recruitment and activation of eosinophils and mast cells, and by stimulating epithelial mucus production, which is known to be mainly dependent on IL-9. These cells share developmental pathways with induced regulatory T cells that may determine the generation of one over the other subset. In fact, the FOXP3 transcription factor has been shown to bind *il9* locus and repress IL-9 production. The microbiota-derived short-chain fatty acids (SCFAs) butyrate and propionate have been described as FOXP3 inducers and are known to have anti-inflammatory properties. While SCFAs attenuate lung inflammation by inducing regulatory T cells and suppressing Th2 responses, their effects on Th9 cells have not been addressed yet. Therefore, we hypothesized that SCFAs would have a protective role in lung inflammation by negatively modulating differentiation and function of Th9 cells. Our results demonstrated that butyrate is more effective than propionate in promoting FOXP3 expression and IL-9 repression. In addition, propionate was found to negatively impact *in vitro* differentiation of IL-13-expressing T cells. Butyrate treatment attenuated lung inflammation and mucus production in OVA-challenged mice, which presented lower frequency of lung-infiltrated Th9 cells and eosinophils. Both Th9 cell adoptive transfer and IL-9 treatment restored lung inflammation in butyrate-treated OVA-challenged mice, indicating that the anti-inflammatory effects of butyrate may rely on suppressing Th9-mediated immune responses.

## Introduction

Helper T-cell differentiation takes place in secondary lymphoid organs, where the cytokine environment plays a key role in determining the fate of naïve T cells toward many CD4+ T-Cellsubsets. These subsets include effector T helper 1 (Th1), Th2, Th9, and Th17 cells that secrete signature cytokines, and regulatory Foxp3 T (Treg) cells that help hold immune responses in check (1). Interleukin-4 (IL-4) is known to induce the differentiation of Th2 cells, characterized by expressing the transcription factors STAT6 and GATA-3, and by producing the cytokines IL-4, IL-5, and IL-13 (1). These cells are mainly involved in the pathogenesis of allergic diseases, promoting activation of eosinophils and mast cells, mucus production, and triggering antibody class switching to IgE in B cells, which can also be modulated by other T-cell subsets (2).

IL-9-producing T cells, which share developmental pathways with both Th2 and Treg cells, are differentiated in response to IL-4, but only when transforming growth factor-β (TGF-β) is present (3). These cells are characterized by expressing the transcription factors PU.1, STAT6, and IRF4, although other molecules are required for IL-9 production, such as BATF and FOXO1 (4–8). STAT6 is particularly important in cellular polarization as it contributes to repressing TGF-β-mediated expression of FOXP3, known to be an IL-9 suppressor (5). Th9 cells are the main cellular source of IL-9, but also produce other cytokines, such as IL-3, IL-10, and IL-21 (3). While described as important effectors against helminthic infections (9, 10), the role of Th9 cells in many inflammatory diseases, cancer, and allergies has been increasingly studied (11–14).

Th9 cells are found in the draining lymph nodes and respiratory tract, especially during early phases of asthma development (15). The peripheral mononuclear blood cells (PBMC) of atopic infants and adults are featured by a higher frequency of Th9 cells when compared to healthy individuals (15, 16), while single nucleotide polymorphisms (SNPs) in the genes encoding both IL-9 and its receptor (IL9R) have been associated with beneficial outcomes in allergen sensitization (17). Experimental data on the role of Th9 cells in lung inflammation have revealed that it mainly relies on promoting IL-9-dependent eosinophil and mast cell infiltration, mucus production and enhanced release of Th2 cytokines (18). In fact, transgenic expression of IL-9 is sufficient to cause bronchial hyperresponsiveness (19, 20), while antibody-mediated neutralization of this cytokine protects mice from inflammation (8). PU.1, IRF4, and BATF knockout mice present impaired differentiation of Th9 cells and lower inflammation (4, 6, 8). On the other hand, thymic stromal lymphopoietin (TSLP) and nitric oxide-mediated activation of these cells was shown to promote increased IL-9 production and disease exacerbation (21, 22).

Although palliative treatments have reduced the severity of symptoms and death of asthma patients, there is no current therapeutic regimen that can cure the disease. A better understanding of the mechanisms involved in inflammation is likely to provide important cues on how to improve treatment and even cure asthma. In this context, an increasing interest in determining the role of the microbiota and their metabolites during disease development has arisen (23).

Mucosal organs such as gut and lungs harbor a large collection of bacteria and other microorganisms that shape local and distal immune responses (23, 24). Birth mode (vaginal or via cesarean section), diet, use of antibiotics and the surrounding environment represent some of the factors that influence microbiota diversity, which is suggested to play an important role in asthma susceptibility (23). Early life differential colonization may have a determinant role in this process and airway microbial diversity appears to be inversely associated with sensitization to house dust mites in childhood (25, 26).

Experimental data on germ-free (GF) mice have demonstrated that lack of microbiota results in stronger ovalbumin (OVA)-induced type 2 airway inflammation and hypersensitivity, which can be reversed by co-housing GF mice with specific pathogen-free (SPF)-raised animals (27). In line with these observations, it has also been shown that antibiotic-mediated depletion of the microbiota renders mice more susceptible to lung inflammation (28). However, not only the microorganisms but also their metabolites have been indicated as key players in controlling immune responses. In fact, microbiota metabolism of dietary fibers and production of short-chain fatty acids (SCFA), such as propionate and acetate, has been considered a major mechanism by which microorganisms control airway inflammation. Induction of Tregs and dendritic cells with impaired capability of promoting effector Th2 responses were shown to play an important role in attenuating disease (29, 30). Nevertheless, the role of SCFAs on other T-cell subsets that participate in lung inflammation, such as Th9 cells, is yet to be addressed.

Given the well-established role of butyrate and propionate in promoting FOXP3 expression during differentiation of Tregs (31, 32) and that FOXP3 is a potent IL-9 repressor (5, 33), we hypothesized that these SCFAs would have a negative impact on the development and function of Th9 cells. We confirmed previous data by showing that butyrate and propionate promote higher FOXP3 expression in the context of Treg differentiation. We then demonstrated an inverse correlation between IL-9 and FOXP3 expression when CD4+ T cells were exposed *in vitro* to butyrate and propionate early during differentiation into Th9 cells. Butyrate was found to be more efficient than propionate in promoting FOXP3 expression and IL-9 repression. In addition, we demonstrated an opposite effect of butyrate and propionate on Th2 cells. While *in vitro* butyrate treatment was responsible for inducing a small, but significant increase in the frequency of IL-13+ T cells, propionate treatment negatively impacted the same cells. Moreover, we found that butyrate-treated OVA-challenged mice presented lower frequency of lung-infiltrated Th9 cells and attenuated inflammation, represented by lower frequency of lung-infiltrated eosinophils, less inflammatory infiltrates and lower mucus production. Adoptive transfer of OVA-specific Th9 cells and recombinant IL-9 treatment were both sufficient to restore lung inflammation in butyrate-treated mice, indicating that butyrate-mediated effects were likely to be dependent on suppression of Th9 cells.

## Materials and Methods

### Animals and Ethics Statement

Male C57BL/6, FOXP3 GFP, and OT-II mice (6–8 weeks old) were obtained from the animal facility of the Institute of Biomedical Sciences, University of São Paulo. Animals were housed in groups of up to 5 per cage in a light- and temperature-controlled room (12 h light/dark cycles, 21 ± 2°C) with free access to food and water. This study was carried out in accordance with the recommendations of the National Institute of Health, Guide for the Care and Use of Laboratory Animals and the Brazilian National Law (11.794/2008). The protocol was approved by the Institutional Animal Care and Use Committee (CEUA) of the University of São Paulo, under protocol number 2015/006.

### OVA-Induced Lung Inflammation

Male C57BL/6 mice were intraperitoneally (IP) injected with 30 μg of ovalbumin (OVA) grade V (Sigma) dissolved in Imject Alum (1.6 mg) (Thermo Fisher), diluted in 200 μl of PBS at days 0 and 7. OVA-sensitized mice were nebulized with an OVA-distillated water solution (3%), using an ultrasonic nebulizer device (Respira Max®) for 15 min at days 14, 15, and 16. Control mice were sensitized as described and nebulized with water only. Mice were euthanized 24 h after the last nebulization (challenge) and lungs were extracted for further analysis.

### Butyrate Treatment

Male C57BL/6 mice were IP injected with 250 μl of 1M butyric acid (butyrate) (Sigma) diluted in PBS (pH: 7.2) or PBS only at days 0, 1, 2, 7, 8, and 9 of OVA-sensitization. Mice treated during sensitization also received butyrate (IP) or PBS during the 3 days of OVA-nebulization (challenge).

### IL-9 Treatment and T Cell Adoptive Transfer

OVA-sensitized butyrate-treated mice were intraperitoneally injected with 150 ng of recombinant murine IL-9 (R&D Systems) diluted in 200 μl of PBS or PBS only at days 1 and 2 of OVA nebulization. Alternatively, butyrate-treated mice were intraperitoneally injected with 2 × 10^6^ OT-II Th0, Th2, or Th9 cells the day before OVA nebulization. OT-II Th2 and Th9 cells were differentiated *in vitro* as described in T cell differentiation topic.

### Lung Digestion and Flow Cytometry

Mice were euthanized and lungs collected, washed in ice-cold PBS, cut in small pieces and incubated in R-10 medium [RPMI-1640 (Thermo Fisher) supplemented with 10% FBS (Thermo Fisher), 2 mM L-glutamine (Thermo Fisher), 1 mM sodium pyruvate (Thermo Fisher), 1% non-essential amino acids (Thermo Fisher), 1% Pen/Strept (Thermo Fisher), 1% vitamin solution (Thermo Fisher), and 5 × 10^−5^ M 2β-mercaptoetanol (Sigma)] containing 0.5 mg/ml of collagenase IV (Thermo Fisher) and 30 μg/ml of DNAse (Sigma), at 37°C for 45 min and 180 rpm. Digested tissues were passed through 100 μm cell strainers (Sigma) and centrifuged. Pellets were resuspended in 1 ml of ACK buffer (Thermo Fisher) for 2 min, centrifuged and resuspended in R-10 medium for further analysis. Cells extracted from lungs were stimulated with PMA (Sigma) 50 ng/ml, ionomycin (Sigma) 500 ng/ml, and brefeldin A (Biolegend) 5 μg/ml for 4 h at 37°C and 5% CO_2_. T cell surface staining was performed for 30 min at 4°C using the following antibodies diluted in PBS: anti-CD45 PercP (BD Biosciences), anti-CD4 APCCy7 (Biolegend) and anti-CD8 FITC (Biolegend). Cells were then fixed and permeabilized using the Cytofix/Cytoperm kit (BD Biosciences). Intracellular staining was performed for 30 min at 4°C with the following antibodies diluted in Perm/wash buffer (BD Biosciences): anti-CD3 APC (BD Biosciences), anti-IL-9 PE (Biolegend), and anti-IL-13 PECy7 (Biolegend). To evaluate lung-infiltrated Tregs, cells were surfaced stained for 30 min at 4°C using the following antibodies diluted in PBS: anti-CD45 PercP (BD Biosciences), anti-CD3 APC (BD Biosciences), and anti-CD4 APCCy7 (Biolegend). FOXP3 expression was determined using the FOXP3/Transcription Factor Staining Buffer Set (eBioscience) with anti-FOXP3 PE (Biolegend), according to the manufacturer's instruction. To evaluate lung-infiltrated eosinophils, cells were surfaced stained for 30 min at 4°C using the following antibodies diluted in PBS: anti-CD45 PercP (BD Biosciences), anti-CD11b APCCy7 (Biolegend), anti-CD11c BV421 (Biolegend), anti-CD64 PE (Biolegend), anti-Ly6G FITC (BD Biosciences), and anti-Siglec F Alexa 647 (BD Biosciences). All samples were acquired with a FACS Canto II (BD Biosciences) and analyzed using FlowJo software (version 9.0.2, Tree Star).

### T Cell Differentiation

Splenocytes from FOXP3 GFP mice were surfaced stained for 30 min at 4°C using the following antibodies diluted in PBS: anti-CD4 PercP (BD Biosciences), anti-CD44 PECy7 (Biolegend) and anti-CD62L APC (BD Biosciences). Naïve CD4+ T cells (CD62L+CD44^low^) were sorted using a FACS Aria III (BD Biosciences) and cultured in R-10 medium in 96-well flat bottom plates (Sigma) with plate bound anti-CD3 (2 μg/ml) (BD Biosciences) and soluble anti-CD28 (0.5 μg/ml) (BD Biosciences) in the presence of Th9 [TGF-β 2 ng/ml (R&D Systems), IL-4 10 ng/ml (Peprotech) and anti-IFNγ 10 μg/ml (BD Biosciences)], Th2 [IL-4 10 ng/ml (Peprotech), anti-IL-12 10 μg/ml (BD Biosciences) and anti-IFNγ 10 μg/ml (BD Biosciences)] or Treg [TGF-β 2 ng/ml (R&D Systems) and IL-2 10 ng/ml (Peprotech)] differentiation conditions for 4 days. Butyric and propionic acids (Sigma) diluted in PBS (250 μM–pH: 7.2) were added in the culture at day 0. At day 4, cells were restimulated with PMA (50 ng/ml) (Sigma), ionomycin (500 ng/ml) (Sigma), and brefeldin A (5 μg/ml) (Biolegend) for 4 h at 37°C and 5% CO_2_. Alternatively, naïve CD4+ T cells were incubated in the presence of Th9 differentiation condition for 4 days. The supernatants were removed and fresh R-10 medium added to the wells. The cells were incubated for additional 4 days in the presence or absence of butyric acid (Sigma) diluted in PBS (250 μM–pH: 7.2). At day 8, cells were restimulated with PMA (50 ng/ml) (Sigma), ionomycin (500 ng/ml) (Sigma), and brefeldin A (5 μg/ml) (Biolegend) for 4 h at 37°C and 5% CO_2_. Cells were then fixed and permeabilized using the Cytofix/Cytoperm kit (BD Biosciences). Intracellular staining was performed for 30 min at 4°C with the following antibodies diluted in Perm/wash buffer (BD Biosciences). Th9 and Treg cells: anti-CD3 APCCy7 (BD Biosciences), anti-IL-9 PE (Biolegend), and anti-IL-10 PercPCy5.5 (Biolegend). Th2 cells: anti-CD3 APCCy7 (BD Biosciences), anti-IL-4 PE (Biolegend), anti-IL-5 APC (Biolegend) and anti-IL-13 PECy7 (Biolegend). Samples were acquired with a FACS Canto II (BD Biosciences) and analyzed using FlowJo software (version 9.0.2, Tree Star).

### Histology

Extracted lungs were cut and the left inferior lobes were fixed in 2 ml of 4% buffered formalin at 4°C and embedded into paraffin. Prepared sections (5 μm) were stained with either H&E or PAS reagents using standardized protocols and analyzed with an Eclipse Ti-E microscope (Nikon Instruments Inc.).

### Statistical Analysis

Statistical analysis was carried out with Graph Pad Prism 6.0 Software. Two-way ANOVA followed by Dunnett's multiple comparison test or One-way ANOVA followed by Tukey's multiple comparison test were used for statistical analysis, depending on data comparisons. *P* < 0.05 was considered significant.

## Results

### Butyrate Impairs Th9 Cell Differentiation

**Figure 1:**
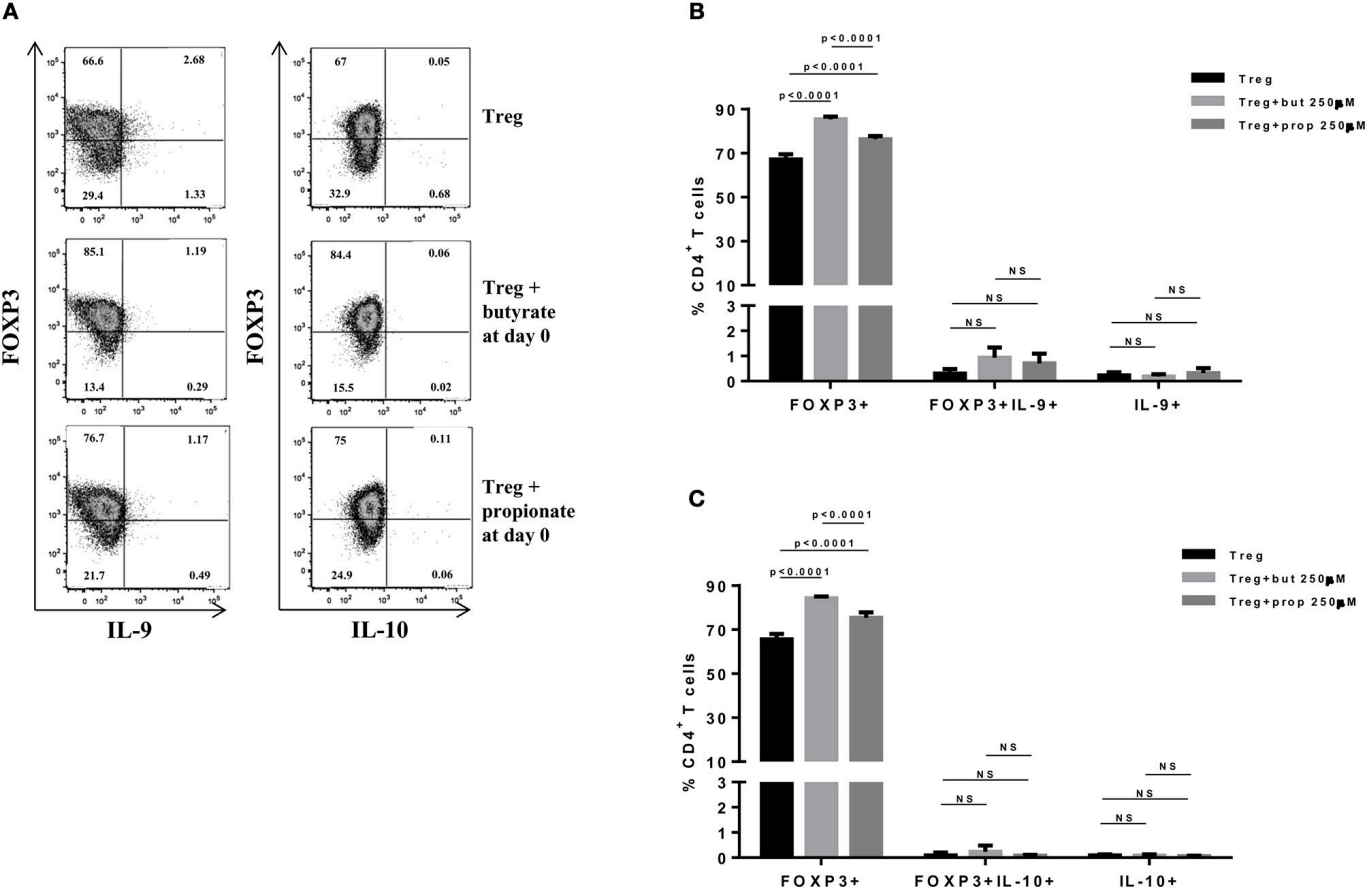
Butyrate and propionate enhances FOXP3 expression in CD4+ T cells differentiated into Tregs. Naïve CD4+ T cells (CD62L+CD44low) were sorted from spleens of FOXP3 GFP mice and cultured in 96-well flat bottom plates with plate bound anti-CD3 (2 μg/ml) and soluble anti-CD28 (0.5 μg/ml) in the presence of Treg (TGF-β 2 ng/ml and IL-2 10 ng/ml) differentiation condition for 4 days. Butyrate and propionate (250 μM) were added in the culture at day 0. At day 4, cells were restimulated with with PMA (50 ng/ml), ionomycin (500 ng/ml), and brefeldin A (5 μg/ml) for 4 h at 37°C and 5% CO_2_, and analyzed by flow cytometry **(A)**. The frequencies of FOXP3+ and/or IL-9+ CD4+ T cells **(B)** and FOXP3+ and/or IL-10+ CD4+ T cells **(C)** are represented by bar graphs. Data are shown as mean ± S.D. Linear regression, Two-way ANOVA and Tukey's multiple comparison test were used for statistical analysis. NS, not significant.

**Figure 2:**
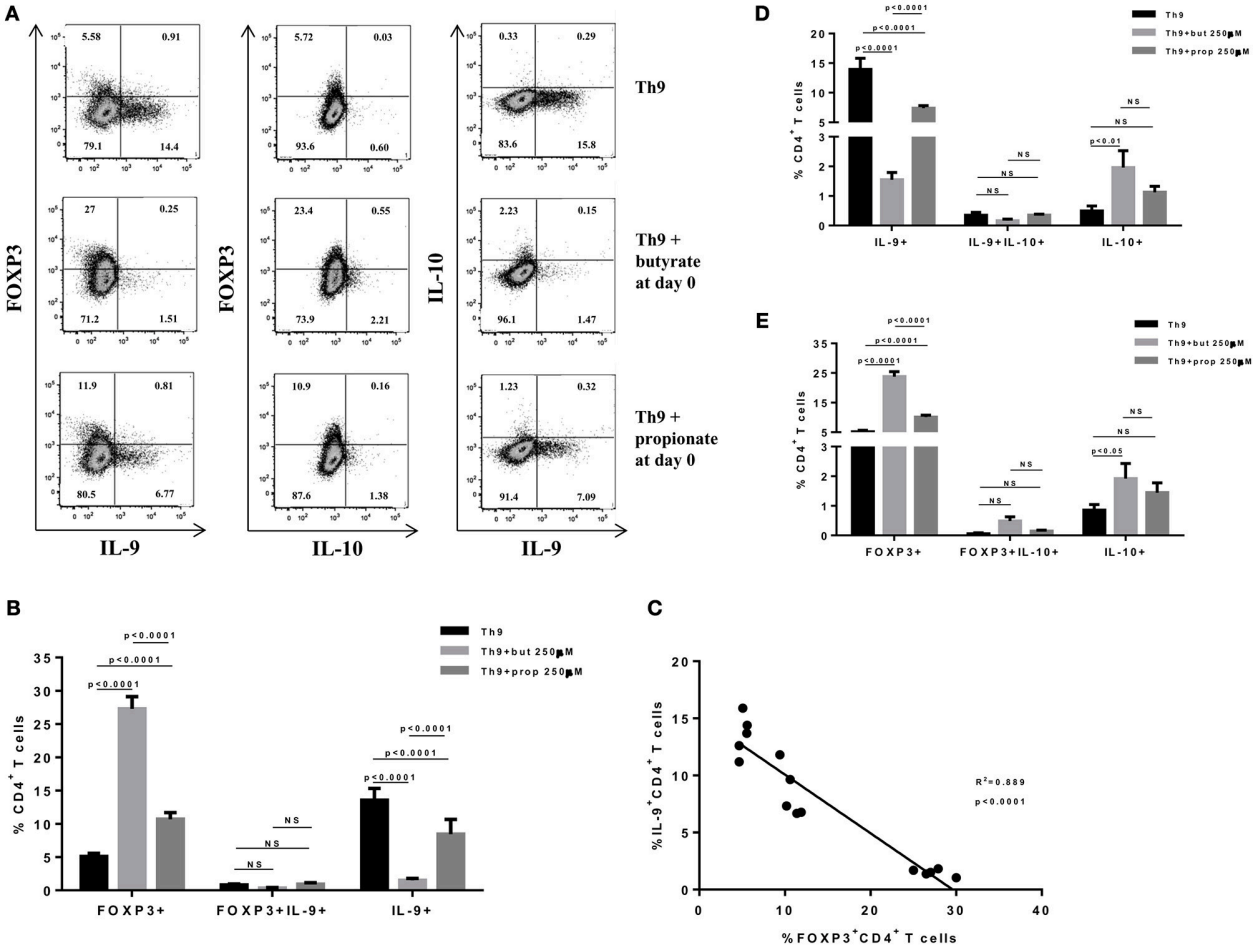
Butyrate enhances FOXP3 expression in CD4+ T cells and impairs the differentiation of Th9 cells. Naïve CD4+ T cells (CD62L+CD44low) were sorted from spleens of FOXP3 GFP mice and cultured in 96-well flat bottom plates with plate bound anti-CD3 (2 μg/ml) and soluble anti-CD28 (0.5 μg/ml) in the presence of Th9 (TGF-β 2 ng/ml, IL-4 10 ng/ml and anti-IFNγ 10 μg/ml) differentiation condition for 4 days. Butyrate and propionate (250 μM) were added in the culture at day 0. At day 4, cells were restimulated with with PMA (50 ng/ml), ionomycin (500 ng/ml), and brefeldin A (5 μg/ml) for 4 h at 37°C and 5% CO_2_, and analyzed by flow cytometry **(A)**. The frequencies of FOXP3+ and/or IL-9+ CD4+ T cells are represented by bar graphs **(B)**. A correlation analysis of IL-9 and FOXP3 expression was performed **(C)**. The frequencies of IL-9+ and/or IL-10+ CD4+ T cells **(D)** and FOXP3+ and/or IL-10+ CD4+ T cells **(E)** were determined and are represented by bar graphs. Data are shown as mean ± S.D. Two-way ANOVA and Tukey's multiple comparison test were used for statistical analysis. NS, not significant.

**Figure 3:**
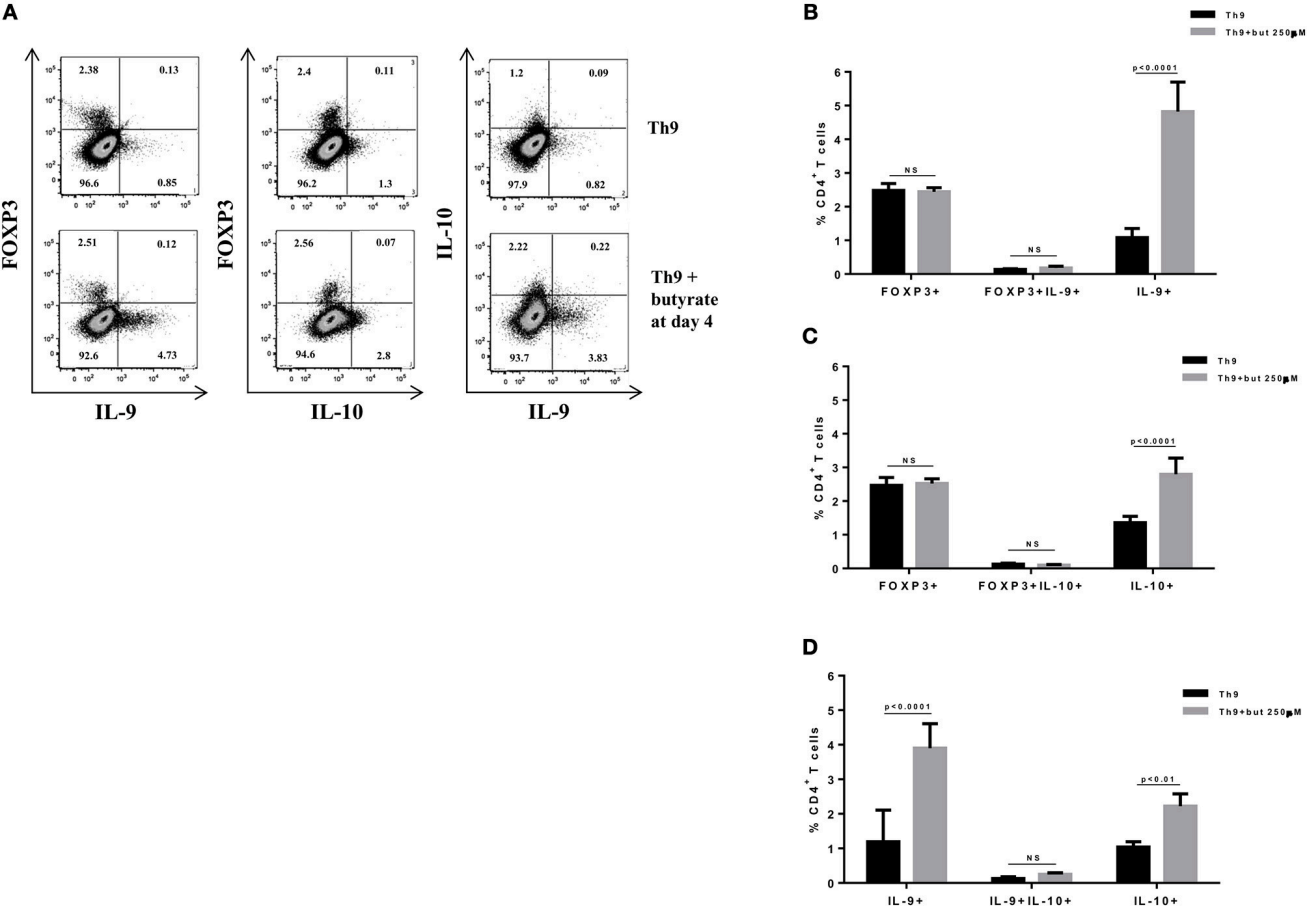
Butyrate sustains cytokine production by differentiated Th9 cells. Naïve CD4+ T cells (CD62L+CD44low) were sorted from spleens of FOXP3 GFP mice and cultured in 96-well flat bottom plates with plate bound anti-CD3 (2 μg/ml) and soluble anti-CD28 (0.5 μg/ml) in the presence of Th9 (TGF-β 2 ng/ml, IL-4 10 ng/ml, and anti-IFNγ 10 μg/ml) differentiation condition for 4 days. At day 4, the medium was replaced by fresh R-10 and butyrate (250 μM) was added in the culture. At day 8, cells were restimulated with with PMA (50 ng/ml), ionomycin (500 ng/ml) and brefeldin A (5 μg/ml) for 4 h at 37°C and 5% CO_2_, and analyzed by flow cytometry **(A)**. The frequencies of FOXP3+ and/or IL-9+ CD4+ T cells **(B)**, FOXP3+ and/or IL-10+ CD4+ T cells **(C)** and IL-9+ and/or IL-10+ CD4+ T cells **(D)** are represented by bar graphs. Data are shown as mean ± S.D. Two-way ANOVA and Sidak's multiple comparison test were used for statistical analysis. NS, not significant.

**Figure 4:**
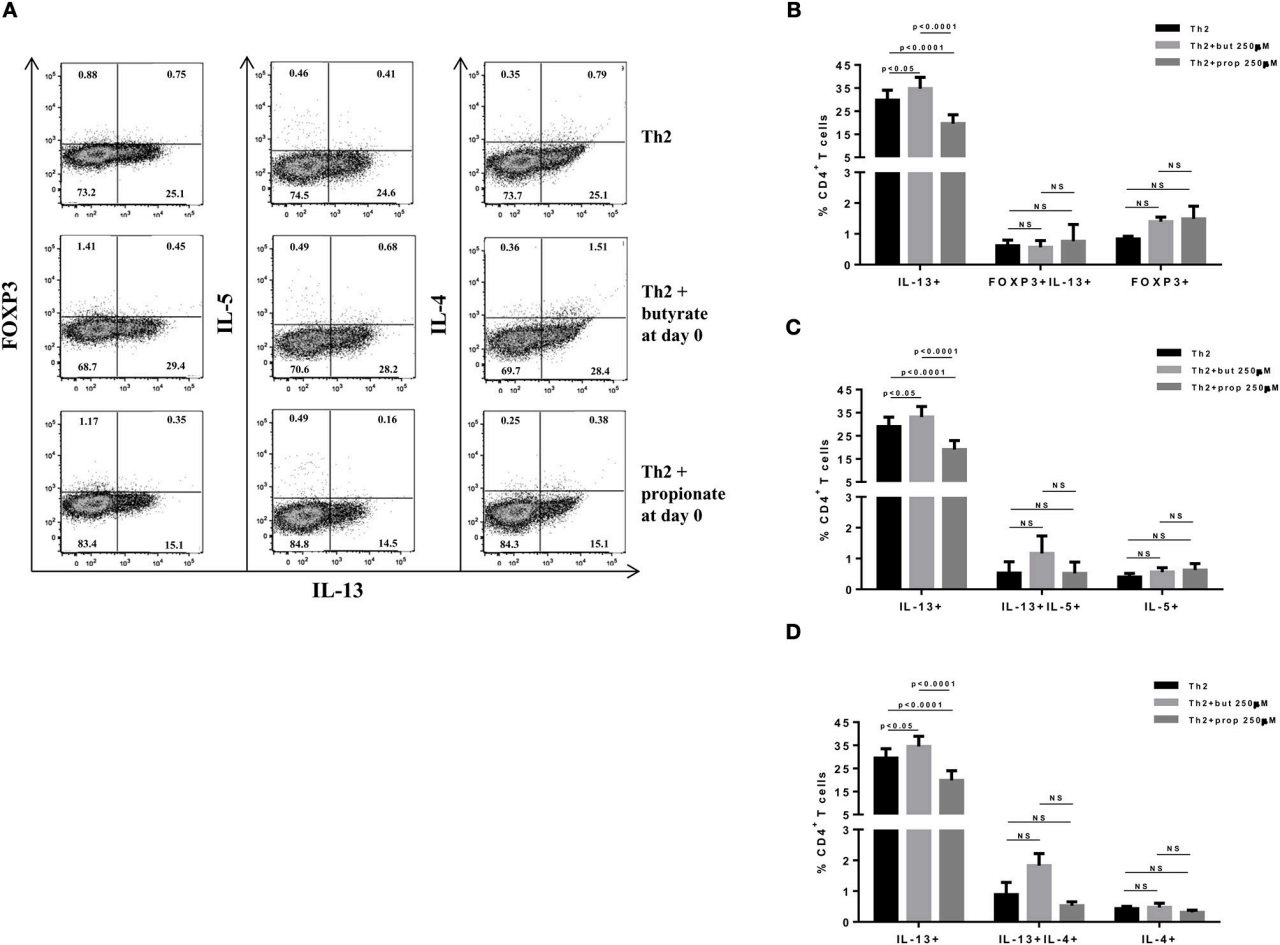
SCFAs have opposite effects on IL-13-expressing T cells. Naïve CD4+ T cells (CD62L+CD44low) were sorted from spleens of FOXP3 GFP mice and cultured in 96-well flat bottom plates with plate bound anti-CD3 (2 μg/ml) and soluble anti-CD28 (0.5 μg/ml) in the presence of Th2 (IL-4 10 ng/ml, anti-IL-12 10 μg/ml, and anti-IFNγ 10 μg/ml) differentiation condition for 4 days. Butyrate and propionate (250 μM) were added in the culture at day 0. At day 4, cells were restimulated with with PMA (50 ng/ml), ionomycin (500 ng/ml), and brefeldin A (5 μg/ml) for 4 h at 37°C and 5% CO_2_, and analyzed by flow cytometry **(A)**. The frequencies of FOXP3+ and/or IL-13+ CD4+ T cells **(B)**, IL-13+ and/or IL-5+ CD4+ T cells **(C)** and IL-13+ and/or IL-4+ CD4+ T cells **(D)** are represented by bar graphs. Data are shown as mean ± S.D. Two-way ANOVA and Tukey's multiple comparison test were used for statistical analysis. NS, not significant.

SCFAs directly modulate the differentiation of CD4+ T cells into Tregs by promoting FOXP3 expression (31), which has been shown to impair IL-9 production (33). Therefore, we first asked whether the SCFAs butyrate and propionate would have any impact on *in vitro* differentiation of Th9 cells. To address this question, naïve CD4+ T cells were sorted from spleens of FOXP3 GFP mice and differentiated into Th9 cells in the presence or absence of butyrate or propionate. Alternatively, naïve CD4+ T cells were differentiated into Tregs and Th2 cells, also in the presence or absence of butyrate or propionate. We confirmed previous data by showing that butyrate and propionate promote FOXP3 expression in the context of Treg differentiation. As expected, butyrate was found to be more efficient in promoting FOXP3 expression than propionate (Figures 1A–C). No significant impact on FOXP3+IL-9+ and FOXP3+IL-10+ T cells was observed (Figures 1A–C). We then demonstrated that butyrate added to the culture at day 0 significantly shifted the cellular differentiation from IL-9-expressing to FOXP3-expressing T cells, while propionate was less effective in promoting the same shift (Figures 2A,B). We found an inverse correlation between IL-9 and FOXP3 expression when all groups were analyzed together (Figure 2C). We also evaluated the impact of butyrate and propionate on IL-10 expression and observed that butyrate treatment resulted in a significantly higher frequency of IL-10-expressing T cells, while IL-10+FOXP3+ and IL-10+IL-9+ T cells were not affected (Figures 2D,E). To further characterize the impact of butyrate on IL-9, IL-10, and FOXP3, we differentiated Th9 cells for 4 days and incubated them for additional 4 days in the presence or absence of butyrate. We found that butyrate treatment had no effect on FOXP3 expression when cells were already differentiated (Figures 3A–C). Most Th9 cells were not expressing IL-9 after 8 days of culture. However, we observed a less intense decrease in the frequency of IL-9-expressing T cells when differentiated Th9 cells were treated with butyrate (Figures 3A,B). No differences were found in FOXP3+IL-9+, FOXP3+IL-10+ and IL-9+IL-10+ T cells (Figures 3B–D). In addition, we demonstrated an opposite effect of butyrate and propionate on Th2 cells. While butyrate was responsible for inducing a small, but significant increase in the frequency of IL-13+ T cells, propionate treatment negatively impacted the same cells. In contrast to Th9 cells, no significant impact on FOXP3 expression was observed in the context of Th2 differentiation (Figures 4A,B). We also evaluated the impact of butyrate and propionate on the frequency of IL-4 and IL-5-expressing T cells and found no significant differences upon treatment (Figures 4C,D). Together, our results demonstrate that butyrate and propionate differently modulate the differentiation of Th9, Th2, and Tregs.

### Butyrate Treatment Negatively Impacts Th9 Cells and Attenuates Lung Inflammation

**Figure 5:**
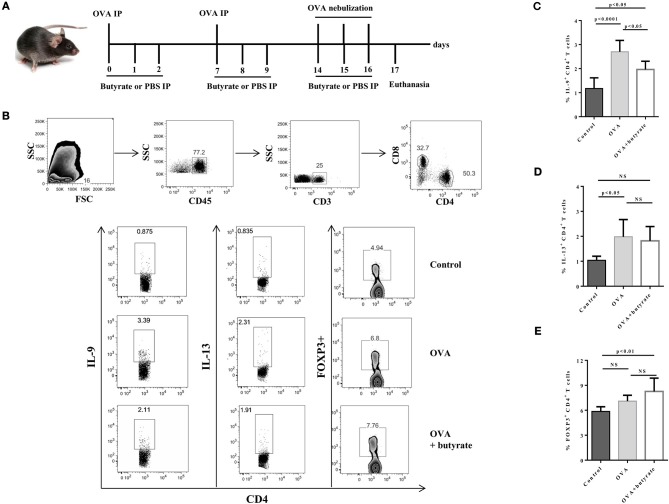
Butyrate treatment negatively impact Th9 cells in the lungs of OVA-challenged mice. Male C57BL/6 mice were intraperitoneally (IP) injected with OVA (30 μg) + Al(OH)3 (1.6 mg) at days 0 and 7. Mice also received 250 μl of either PBS or butyrate (1M) (IP) at days 0, 1, 2, 7, 8, and 9. OVA-sensitized mice were nebulized with an OVA solution (3%) for 15 min at days 14, 15, and 16. The group that received butyrate during sensitization was also treated during challenge. A control group was sensitized and challenged without OVA. Euthanasia was performed 24 h after the last challenge **(A)**. Lungs were digested, cells stimulated with PMA (50 ng/ml), ionomycin (500 ng/ml) and brefeldin A (5 μg/ml) for 4 h at 37°C and 5% CO_2_, and stained with monoclonal antibodies to determine the frequencies of IL-9+, IL-13+, and FOXP3+ CD4+ T cells by flow cytometry **(B)**. Bar graphs show the frequencies of IL-9+ **(C)**, IL-13+ **(D)** and FOXP3+ **(E)** CD4+ T cells in the different groups. Data are shown as mean ± SD. One-way ANOVA followed by Tukey's multiple comparison test were used for statistical analysis. *n* = 5–7 mice per group. NS, not significant.

**Figure 6:**
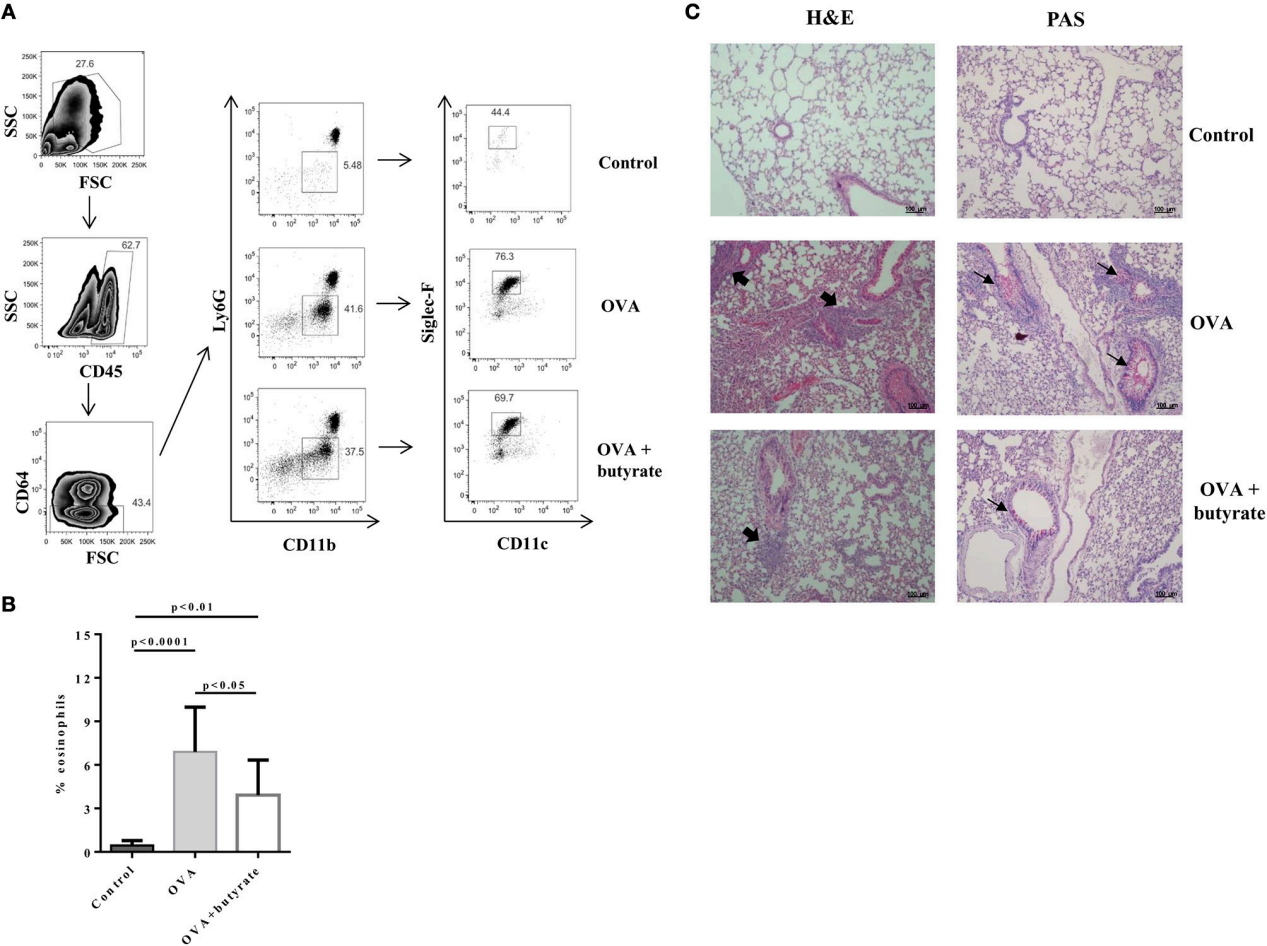
Butyrate treatment protects OVA-challenged mice from lung inflammation. Male C57BL/6 mice were intraperitoneally (IP) injected with OVA (30 μg) + Al(OH)3 (1.6 mg) at days 0 and 7. Mice also received 250 μl of either PBS or butyrate (1 M) (IP) at days 0, 1, 2, 7, 8, and 9. OVA-sensitized mice were nebulized with an OVA solution (3%) for 15 min at days 14, 15, and 16. The group that received butyrate during sensitization was also treated during challenge. A control group was sensitized and challenged without OVA. Euthanasia was performed 24 h after the last challenge. Lungs were digested and cells stained with monoclonal antibodies to determine the frequency of eosinophils (CD45+CD64-Ly6G-CD11b+Siglec-F+CD11c-) by flow cytometry **(A)**. Bar graph shows the frequency of eosinophils in the different groups **(B)**. Lung tissues were also stained with hematoxylin/eosin (H&E) and periodic acid–Schiff (PAS), scale bars: 100 μm. Thick and thin arrows indicate inflammatory infiltrates and mucus production, respectively **(C)**. Data are shown as mean ± SD. One-way ANOVA followed by Tukey's multiple comparison test were used for statistical analysis. *n* = 5–7 mice per group.

Given the *in vitro* effect of butyrate on Th9 cells, we next sought to determine whether it would also have an *in vivo* impact on these cells. To address this question, we induced OVA-mediated lung inflammation in C57BL/6 mice treated or not with butyrate (Figure 5A) and evaluated the frequencies of lung-infiltrated Th9, Th2, and Treg cells by flow cytometry (Figure 5B). We found that butyrate-treated animals presented a significantly lower frequency of lung-infiltrated Th9 cells (Figure 5C) while no difference was observed for IL-13-expressing Th2 cells (Figure 5D). Butyrate-treated OVA-challenged mice had significantly higher lung-infiltrated Treg cells when compared to control, but no statistical significance was found when compared to OVA-challenged mice (Figure 5E). Considering the pre-established role of IL-9 in promoting the recruitment of eosinophils to the lungs and supporting inflammation (34), we further analyzed animal lungs for the presence of eosinophils (CD45+CD64-Ly6G-CD11b+Siglec-F+CD11c-) (Figure 6A) and found a significantly higher frequency of these cells infiltrated in the lungs of OVA-challenged mice when compared to both control and butyrate-treated OVA-challenged mice (Figure 6B). Higher inflammation and mucus production were also observed in OVA-challenged mice when compared to the other groups (Figure 6C). Therefore, our results indicate that butyrate may protect mice from lung inflammation by impairing the differentiation of Th9 cells.

### Adoptive Transfer of Th9 Cells Reverses Butyrate Effects on Lung Inflammation

**Figure 7:**
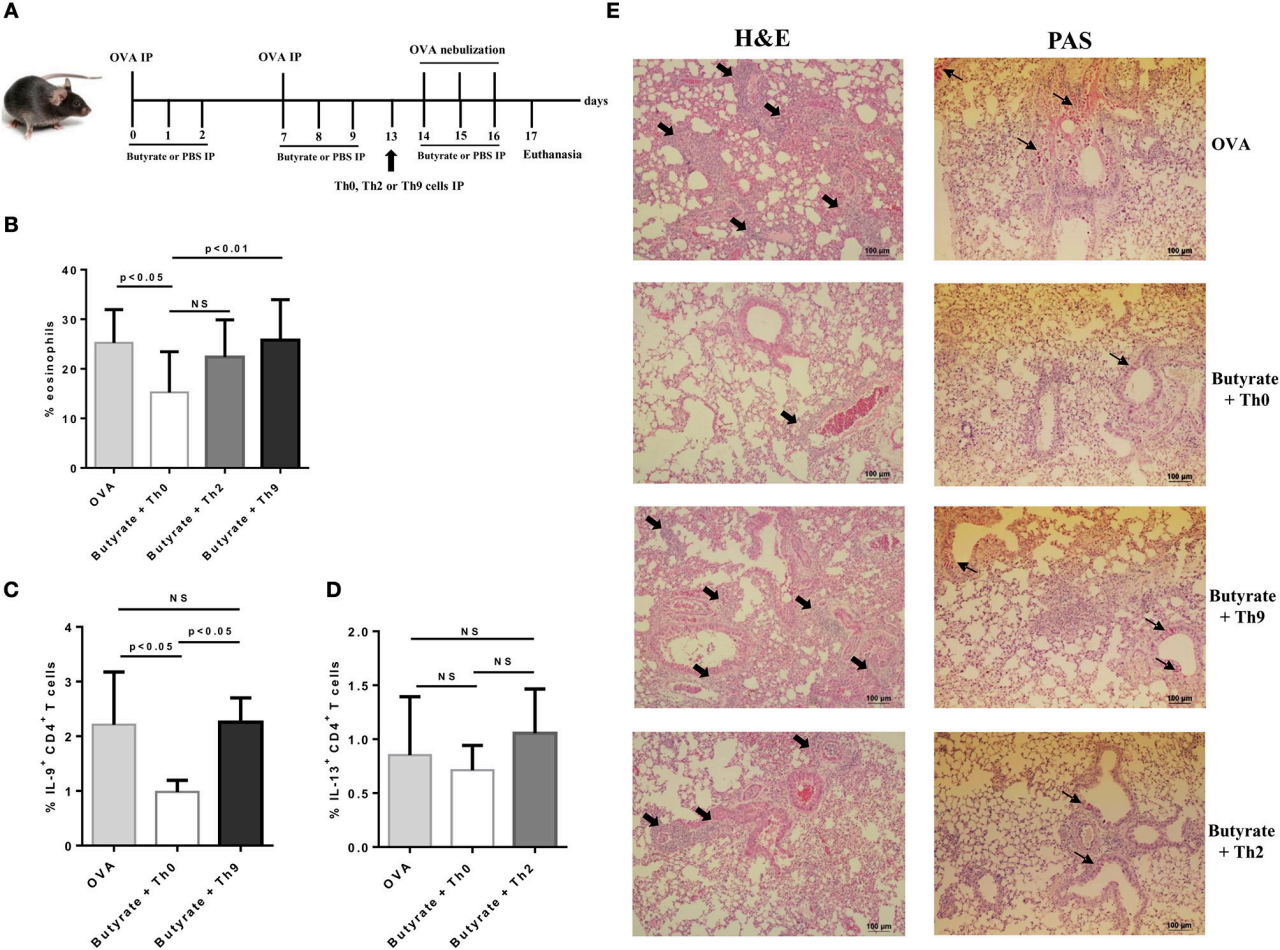
Adoptive transfer of Th9 cells restores lung inflammation in butyrate-treated OVA-challenged mice. Male C57BL/6 mice were intraperitoneally (IP) injected with OVA (30 μg) + Al(OH)3 (1.6 mg) at days 0 and 7. Mice also received 250 μl of either PBS or butyrate (1 M) (IP) at days 0, 1, 2, 7, 8, and 9. OVA-sensitized mice were nebulized with an OVA solution (3%) for 15 min at days 14, 15, and 16. The groups that received butyrate during sensitization were treated during challenge. Butyrate-treated mice also received an adoptive transfer (IP) of OT-II Th0, Th2, or Th9 cells 24 h before challenge initiation. Euthanasia was performed 24 h after the last challenge **(A)**. Lungs were digested and cells stained with monoclonal antibodies to determine the frequency of eosinophils as shown by the bar graph **(B)**. Alternatively, cells were stimulated with PMA (50 ng/ml), ionomycin (500 ng/ml), and brefeldin A (5 μg/ml) for 4 h at 37°C and 5% CO_2_, and stained with monoclonal antibodies to determine the frequency of IL-9+CD4+ and IL-13+CD4+ T cells as shown by the bar graphs **(C,D)**, respectively. Lung tissues were also stained with hematoxylin/eosin (H&E) and periodic acid–Schiff (PAS), scale bars: 100 μm. Thick and thin arrows indicate inflammatory infiltrates and mucus production, respectively **(E)**. Data are shown as mean ± SD. One-way ANOVA followed by Tukey's multiple comparison test were used for statistical analysis. *n* = 5–7 mice per group. NS, not significant.

To confirm that butyrate treatment protected mice from lung inflammation by negatively modulating the differentiation of Th9 cells, we performed OVA-challenge experiments as previously described, in which butyrate-treated mice also received an adoptive transfer of either naïve OT-II Th0 cells or *in vitro*-differentiated OT-II Th2 or Th9 cells one day before initiating OVA-challenge (Figure 7A). We observed that adoptive transfer of Th9 cells restored the frequency of lung-infiltrated eosinophils in butyrate-treated OVA-challenged mice to the same level of mice not treated with butyrate (Figure 7B). Th2 adoptive transfer resulted in a higher frequency of lung-infiltrated eosinophils, although it was not statistically significant (Figure 7B). We also demonstrated that the frequency of lung-infiltrated Th9 cells was restored in animals that received these cells (Figure 7C). Th2 adoptive transfer resulted in a small increase in the frequency of these cells into the lungs of butyrate-treated OVA-challenged mice (Figure 7D). Lung inflammation and mucus production were also higher in animals that received adoptive transfer of Th9 cells, and in a lesser extent in those that received Th2 cells, when compared to butyrate-treated Th0-injected mice (Figure 7E). Taken together, these results reinforce our hypothesis that butyrate treatment attenuates lung inflammation by negatively modulating Th9 cells.

### IL-9 Treatment Counteracts Butyrate Effects on Lung Inflammation

**Figure 8:**
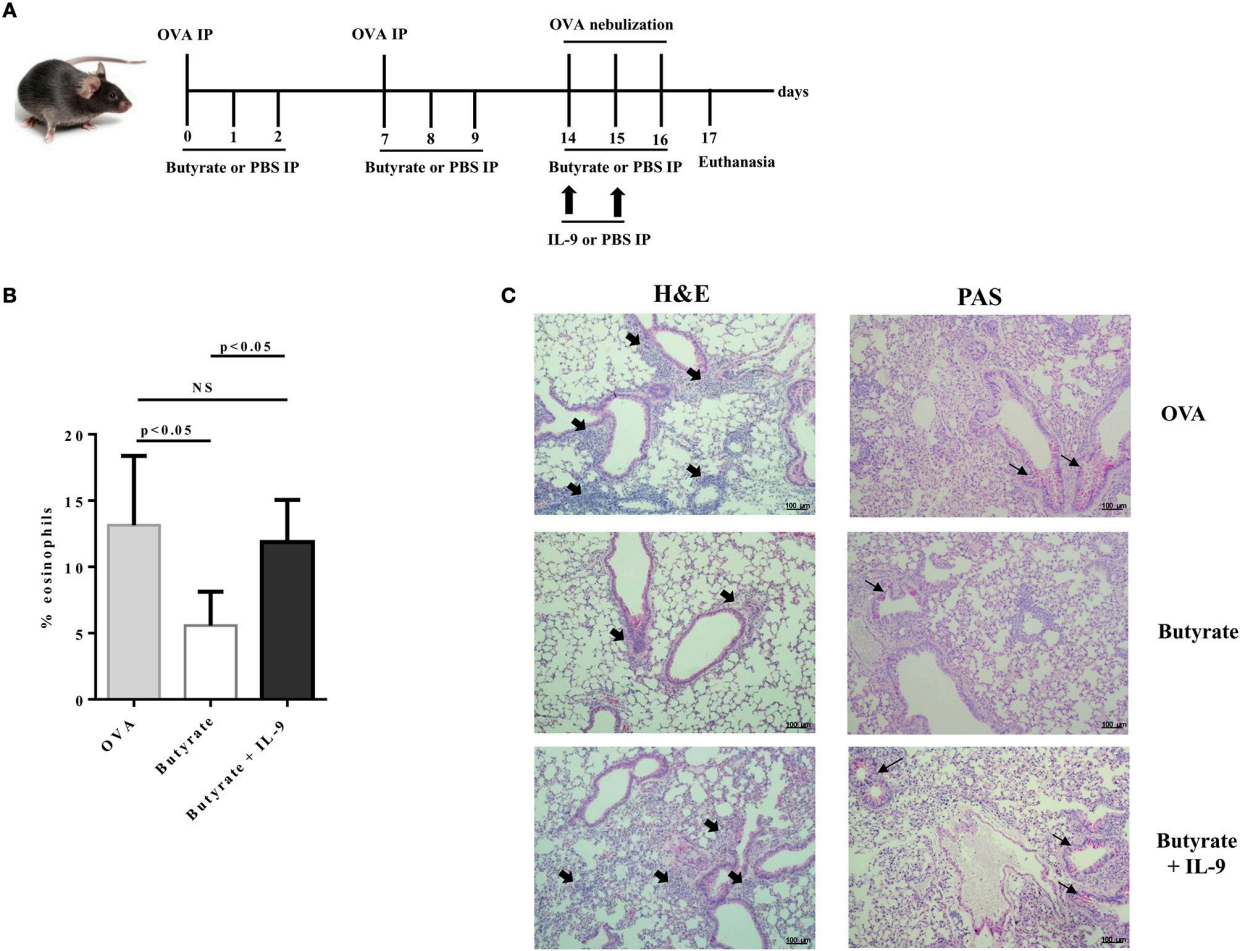
IL-9 treatment restores lung inflammation in butyrate-treated OVA-challenged mice. Male C57BL/6 mice were intraperitoneally (IP) injected with OVA (30 μg) + Al(OH)3 (1.6 mg) at days 0 and 7. Mice also received 250 μl of either PBS or butyrate (1M) (IP) at days 0, 1, 2, 7, 8, and 9. OVA-sensitized mice were nebulized with an OVA solution (3%) for 15 min at days 14, 15, and 16. The groups that received butyrate during sensitization were also treated during challenge. Butyrate-treated mice also received either PBS or recombinant IL-9 (IP) at days 14 and 15. Euthanasia was performed 24 h after the last challenge **(A)**. Lungs were digested and cells stained with monoclonal antibodies to determine the frequency of eosinophils as shown by the bar graph **(B)**. Lung tissues were also stained with hematoxylin/eosin (H&E) and periodic acid–Schiff (PAS), scale bars: 100 μm. Thick and thin arrows indicate inflammatory infiltrates and mucus production, respectively **(C)**. Data are shown as mean ± SD. One-way ANOVA followed by Tukey's multiple comparison test were used for statistical analysis. *n* = 5–7 mice per group. NS, not significant.

**Figure 9:**
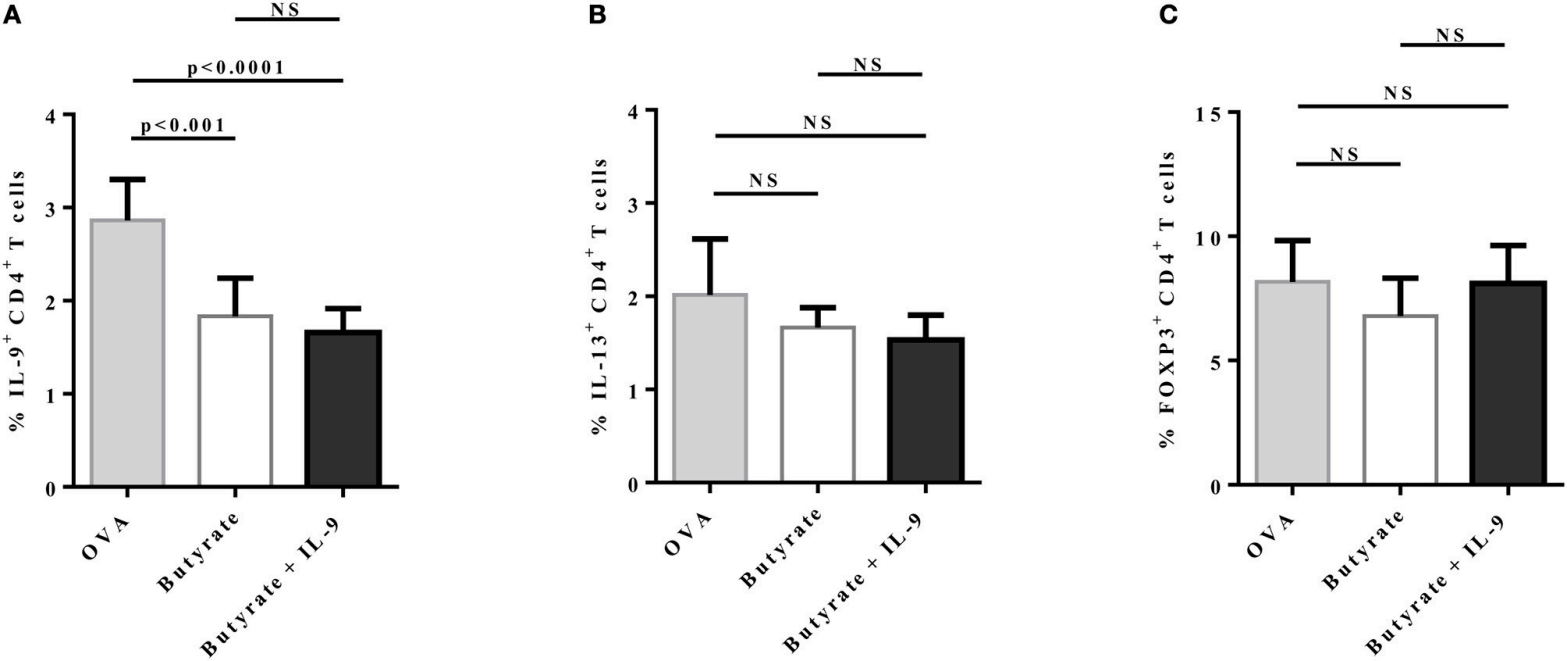
IL-9 treatment has no impact on IL-9+, IL-13+, and FOXP3+ CD4+ T cells in the lungs of butyrate-treated OVA-challenged mice. Male C57BL/6 mice were intraperitoneally (IP) injected with OVA (30 μg) + Al(OH)3 (1.6 mg) at days 0 and 7. Mice also received 250 μl of either PBS or butyrate (1 M) (IP) at days 0, 1, 2, 7, 8, and 9. OVA-sensitized mice were nebulized with an OVA solution (3%) for 15 min at days 14, 15, and 16. The groups that received butyrate during sensitization were also treated during challenge. Butyrate-treated mice also received either PBS or recombinant IL-9 (IP) at days 14 and 15. Euthanasia was performed 24 h after the last challenge. Lungs were digested, cells stimulated with PMA (50 ng/ml), ionomycin (500 ng/ml), and brefeldin A (5 μg/ml) for 4 h at 37°C and 5% CO_2_, and stained with monoclonal antibodies to determine the frequencies of IL-9+ **(A)**, IL-13+ **(B)**, and FOXP3+ **(C)** CD4+ T cells in the different groups. Data are shown as mean ± SD. One-way ANOVA followed by Tukey's multiple comparison test were used for statistical analysis. *n* = 5–7 mice per group. NS, not significant.

To address the question whether butyrate treatment was attenuating lung inflammation by decreasing IL-9 effects, we performed OVA-challenge experiments as previously described, but including a new group of butyrate-treated mice that also received recombinant IL-9 (Figure 8A). We demonstrated that intraperitoneal treatment with IL-9 during OVA-challenge was sufficient to restore the frequency of lung-infiltrated eosinophils in butyrate-treated mice to a similar level of OVA-challenged mice (Figure 8B). We also observed that IL-9 treatment promoted higher inflammation and mucus production in butyrate-treated mice when compared to animals not treated with IL-9 (Figure 8C). We then looked at the frequencies of lung-infiltrated Th9, Th2, and Treg cells and found that IL-9 treatment had no effect on these cells (Figures 9A–C, respectively). Thus, these results indicate that butyrate may exert an anti-inflammatory impact on OVA-challenged mice by reducing IL-9 effects.

## Discussion

In this study, we demonstrate that butyrate is more effective than propionate in promoting FOXP3 expression and IL-9 repression. In addition, we show that propionate negatively impact *in vitro* differentiation of IL-13-expressing T cells. Butyrate treatment attenuated lung inflammation and mucus production in OVA-challenged mice, which presented lower frequency of lung-infiltrated Th9 cells and eosinophils. Both Th9 cell adoptive transfer and IL-9 treatment restored lung inflammation in butyrate-treated OVA-challenged mice, indicating that the anti-inflammatory effects of butyrate may rely on suppressing Th9-mediated immune responses.

Given the increasing evidence that microbiota-derived metabolites contribute to modulating inflammatory diseases such as asthma, colitis and arthritis (29, 30, 35–37), we sought to evaluate whether the SCFAs butyrate and propionate would have an impact on the differentiation of Th9 cells, key players in inflammation (3). We demonstrated that butyrate added to the culture at day 0 significantly shifted the cellular differentiation from IL-9-expressing to FOXP3-expressing T cells, while propionate was less effective in promoting the same shift. A strong inverse correlation between IL-9 and FOXP3 expression was found when all groups were analyzed together. Additional analysis showed that butyrate treatment led to higher frequency of IL-10-expressing T cells that were not expressing either FOXP3 or IL-9. Although we cannot assume that the cells generated by the exposition to butyrate or propionate under Th9 differentiation were converted to induced regulatory T cells (iTregs), we were still able to demonstrate the negative impact of these SCFAs on Th9 cells.

To further characterize the impacts of butyrate on Th9 cells, we treated differentiated cells and found no impact on FOXP3 expression. We found that most Th9 cells were not expressing IL-9 after 8 days of culture, when compared to cells stimulated with PMA after 4 days of differentiation. Surprisingly, we observed a less intense decrease in the frequency of IL-9-expressing T cells when differentiated Th9 cells were treated with butyrate, suggesting that this SCFA may have opposite effects on naïve CD4+ T cells and differentiated Th9 cells. A higher frequency of IL-10-expressing T cells was also found when differentiated Th9 cells were exposed to butyrate. A recent study demonstrated that butyrate promotes IL-10 expression by differentiated Th1 cells through interaction with the GPR43 receptor (38), supporting our findings. Although we also observed higher frequency of IL-9-expressing T cells when Th9 cells were treated with butyrate, no impact on IFN-γ-expressing T cells was demonstrated in the previous report, suggesting that the effector cytokines produced by Th9 and Th1 cells are differentially affected by butyrate.

The effects of butyrate and other SCFAs on FOXP3 expression in CD4+ T cells differentiated into iTregs have been previously demonstrated (31, 32), as we have also shown here. It is known that Th9 cells and iTregs share key transcription factors and common induction pathways that may determine the generation of one over the other subset. While GITR engagement was shown to subvert iTregs differentiation by favoring Th9 cells, FOXP3-mediated repression of the *Il9* locus contributed to blocking the differentiation of Th9 cells (33). Therefore, we believe that the negative impact of butyrate on Th9 cells are likely to be dependent on FOXP3-mediated repression of IL-9 early during differentiation, as we have not found the same effect when exposing differentiated cells to butyrate. This SCFA could also be acting through epigenetic remodeling of the *Il9* locus (39) or by breaking down signal pathways involved in IL-9 expression. Studies with FOXP3-deficient cells could be performed to further confirm the role of FOXP3 in mediating butyrate-induced IL-9 repression.

To further investigate the effects of butyrate on Th9 cells, we established an OVA-induced lung inflammation model and demonstrated that butyrate treatment resulted in lower frequency of lung-infiltrated Th9 cells, while no impact on Th2 cells was observed. Higher frequency of lung-infiltrated FOXP3+ T cells was found in butyrate-treated OVA-challenged mice when compared to control animals, but no significant differences were found when compared to OVA-challenged mice. Acetate-mediated protection against house dust mite extract (HDM)-induced lung inflammation has been previously reported, indicating that SCFAs may act through induction of iTregs to inhibit inflammation. However, a 3 week-treatment period was necessary for the observed results (30). In our experiments, animals were treated for a much shorter period, which could explain the lack of significant difference in terms of lung-infiltrated FOXP3+ T cells between OVA-challenged groups. As regulatory T cells naturally migrate to inflamed tissues to suppress inflammation (40), it is possible that butyrate-mediated induction of iTregs was masked in OVA-challenged mice due to the increased influx of these cells into the lungs, independently of their origin. On the other hand, iTregs generated in the gut upon intraperitoneal butyrate treatment may have not appropriately responded to the lung OVA challenge, resulting in less lung infiltration than expected. The *in vivo* instability of iTregs (41) could also be an explanation for not having a significant increase in lung infiltration upon butyrate treatment. These cells may have lost FOXP3 expression during the sensitization period. Therefore, it is unlikely that induction of iTregs played a major role in our study, although our *in vitro* experiments suggest an inverse correlation of FOXP3 and IL-9 expression upon treatment with SCFAs.

Lung-infiltrated eosinophils were used as a parameter to evaluate Th9-mediated immune responses in our model, given the well-established role of IL-9 in promoting eotaxin/CCL11 expression and influx of these cells into different body tissues, including lungs (34, 42, 43). We found that butyrate-treated OVA-challenged mice presented lower frequency of lung-infiltrated eosinophils, attenuated inflammatory infiltrates and mucus production, indicating that Th9-mediated immune responses were suppressed. In line with our observations, a previous study has demonstrated that dietary fiber and the SCFA propionate protected mice from lung inflammation and asthma symptoms. However, the effects of propionate relied on promoting dendritic cells with impaired capability of inducing effector Th2 cells in an HDM-mediated model. Significant differences in the immunological parameters of propionate-treated mice were observed 5–6 days after the last challenge (29). In our study, we have found a more acute effect of butyrate, with significant differences 24 h after the last challenge, suggesting that these SCFAs may have different impacts on the immune system in the context of lung inflammation.

Although we have demonstrated that *in vitro* butyrate treatment induced a small, but significant increase in the frequency of IL-13-expressing T cells, no *in vivo* effects on these cells were observed, indicating that Th9-mediated immune responses were likely to be the most affected in our model. As we have not found a significant *in vitro* impact of butyrate on the frequency of IL-4 and IL-5-expressing T cells, these cytokines were not evaluated in our *in vivo* experiments. However, to further characterize the effects of SCFAs on Th2 cells, we also treated naïve CD4+ T cells with propionate and demonstrated that it partially impaired the differentiation of IL-13-expressig T cells while not affecting IL-4 and IL-5. We understand that more studies are still necessary to unveil the mechanisms involved in this phenomenon, but conceive that our results complement the previous report on the impact of propionate on Th2 cells.

To confirm that butyrate was attenuating lung inflammation by suppressing Th9 cells, we performed either adoptive transfer of OVA-specific OT-II Th9 cells or recombinant IL-9 treatment and found that both strategies were sufficient to restore lung inflammation in butyrate-treated OVA-challenged mice. Adoptive transfer of OT-II Th2 cells also increased lung inflammation, but to a lesser extent than that observed with Th9 transfer. Although it has been shown that IL-9 may promote IL-13-mediated lung inflammation and mucus production (20), we found no difference in the frequency of lung-infiltrated IL-13-expressing Th2 cells in IL-9-treated mice, suggesting that other cells may have mediated the inflammatory process. In fact, type II innate lymphoid cells (ILC2) are known to produce IL-13 and to play a role in experimental and clinical asthma pathology (44, 45). A synergistically effect of ILC2 and Th9 cells in lung inflammation has also been described (46, 47), suggesting that ILC2 may have a role in our model. On the other hand, our results could be partially explained by the fact that IL-9 exerts a direct effect on lung epithelial cells and promotes mucus production (48). We cannot rule out the possibility that butyrate directly impacted ILC2 and even other cell populations, as recently reported (49). However, our data strongly suggest that Th9/IL-9 played a major role in our model. Further studies are still necessary to mechanistically confirm our hypothesis.

Our findings bring new insights on the mechanisms by which microbiota-derived metabolites regulate diseases, suggesting that butyrate attenuates lung inflammation most likely through a negative modulation of Th9 cell-mediated immune responses. Therefore, butyrate should be clinically considered in the treatment of inflammatory diseases in which suppression of Th9 cells is necessary.

## Author Contributions

RA and RV conceived and designed the experiments. RV, AC, and PB performed and analyzed experiments. RA and RV prepared figures and wrote the manuscript. RA and NC performed final review of the manuscript. NC provided structure and reagents. All authors read and approved the final article.

### Conflict of Interest Statement

The authors declare that the research was conducted in the absence of any commercial or financial relationships that could be construed as a potential conflict of interest.
